# Windfalls and pitfalls: Applications of population genetics to the search for disease genes

**DOI:** 10.1093/emph/eot021

**Published:** 2013-11-06

**Authors:** Michael D. Edge, Prakash Gorroochurn, Noah A. Rosenberg

**Affiliations:** ^1^Department of Biology, Stanford University, Stanford, CA 94305-5020, USA and ^2^Department of Biostatistics, Mailman School of Public Health, Columbia University, New York, NY 10032, USA

**Keywords:** genome-wide association studies, imputation, linkage disequilibrium, population stratification

## Abstract

Association mapping can be viewed as an application of population genetics and evolutionary biology to the problem of identifying genes causally connected to phenotypes. However, some population-genetic principles important to the design and analysis of association studies have not been widely understood or have even been generally misunderstood. Some of these principles underlie techniques that can aid in the discovery of genetic variants that influence phenotypes (‘windfalls’), whereas others can interfere with study design or interpretation of results (‘pitfalls’). Here, considering examples involving genetic variant discovery, linkage disequilibrium, power to detect associations, population stratification and genotype imputation, we address misunderstandings in the application of population genetics to association studies, and we illuminate how some surprising results in association contexts can be easily explained when considered from evolutionary and population-genetic perspectives. Through our examples, we argue that population-genetic thinking—which takes a theoretical view of the evolutionary forces that guide the emergence and propagation of genetic variants—substantially informs the design and interpretation of genetic association studies. In particular, population-genetic thinking sheds light on genetic confounding, on the relationships between association signals of typed markers and causal variants, and on the advantages and disadvantages of particular strategies for measuring genetic variation in association studies.

Identification of genetic loci that contribute to phenotypic outcomes is one of the most important missions of genetics. Researchers continue to vigorously seek genetic factors that underlie disease phenotypes and other traits, particularly in humans. Over the last decade, a primary tool in this search has been the association study, which aims to locate disease-susceptibility loci by identifying alleles whose presence in study subjects is statistically associated with the occurrence of disease. Association studies rely on the fact that once disease variants arise, evolutionary processes including mutation, recombination and coalescence of genealogical lineages tend to maintain associations between susceptibility alleles and disease status and to disrupt associations between disease status and other alleles. Thus, the utility of association mapping for locating disease loci is grounded in population-genetic phenomena.

Association-based identification of disease-susceptibility loci is difficult partly because associations between genotypes and phenotypes are complicated by the evolutionary histories and population-genetic properties of the genomic regions under study. Depending on the specific populations and study designs used, these complications can lead to failures to uncover true causal relationships, attributions of true signals to the wrong markers and detections of spurious associations that are not due to any causal relationship. At the same time, other aspects of evolutionary and population-genetic thinking provide a basis for surprising techniques that enhance the overall program of association mapping. In some cases, difficulties and successes in the application of population genetics to association mapping are widely appreciated and are routinely incorporated in study design and analysis. In other cases, however, mechanisms by which population-genetic processes hinder or aid association studies are counterintuitive or not generally recognized.

To facilitate a deeper understanding of association studies and their basis in evolutionary biology and population genetics, this article aims to illuminate some recurring difficulties or misunderstandings (‘pitfalls’) and some unexpected successes (‘windfalls’) in the application of population genetics to association studies.

## 1. PITFALL: THE MAXIMUM POSSIBLE VALUE OF THE *R*^2^ STATISTIC FOR LINKAGE DISEQUILIBRIUM BETWEEN TWO LOCI IS TYPICALLY NOT 1. INSTEAD, IT IS A FUNCTION OF THE ALLELE FREQUENCIES OF THE LOCI

In the 1990s, well before the advent of genome-wide association (GWA) studies, linkage disequilibrium (LD) was increasingly recognized as a potentially valuable tool for genetic mapping [1, 2]. Given that testing every locus in the human genome for disease association was not imminently feasible, the idea of indirect association testing arose, in which a subset of markers carefully chosen to ‘capture’ ungenotyped variation would be examined [3, 4]. These markers would not necessarily be expected to have a direct impact on disease risk; rather, associations between a marker and disease would represent statistical associations arising from LD between genotyped markers and true causal loci. The measurement of LD—a topic that originated in evolutionary modeling [5, 6] and had long been of interest in purely population-genetic studies [7–9]—became fundamental to the dominant paradigm for genetic mapping. Among the LD measures available [1], the squared correlation coefficient *r*^2^ emerged as particularly useful in association mapping because of its simplicity, its interpretation as the square of a correlation, and its relationship to the power to detect association with a marker in LD with a causal variant—but more on that later. As a consequence of this important new role for *r*^2^, mathematical properties of the *r*^2^ statistic have become directly relevant to association mapping. Some of these properties have not always been understood, potentially leading to confusion in the interpretation of association studies.

As a general rule, correlation coefficients lie between −1 and +1, and the same is true in the LD context for the correlation coefficient *r*, which measures the correlation between two indicator variables, one for the presence of a specific allele at the first of a pair of biallelic loci and the other for the presence of a specific allele at the second locus. It is tempting to assume that *r* ranges from −1 to 1 (and thus *r*^2^ ranges from 0 to 1) for any pair of markers with any allele frequencies. This is a misunderstanding.

Squared correlations between binary indicator variables, in fact, can only achieve a value of 1 when the variables have equal expectation or when the sum of their expectations is 1. Otherwise, the upper bound is strictly smaller than 1. Consider two loci, one with alleles *A* and *a* and the other with alleles *B* and *b*, where *p_A_*, *p_a_* = 1 − *p_A_*, *p_B_*, and *p_b_* = 1 − *p_B_* represent the (non-zero) frequencies of alleles *A*, *a*, *B* and *b*, respectively, and *p_AB_*, *p_Ab_*, *p_aB_* and *p_ab_*, respectively, represent the frequencies of haplotypes *AB*, *Ab*, *aB* and *ab*. Denoting the correlation coefficient between the two loci by

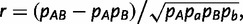

**Figure 1.**
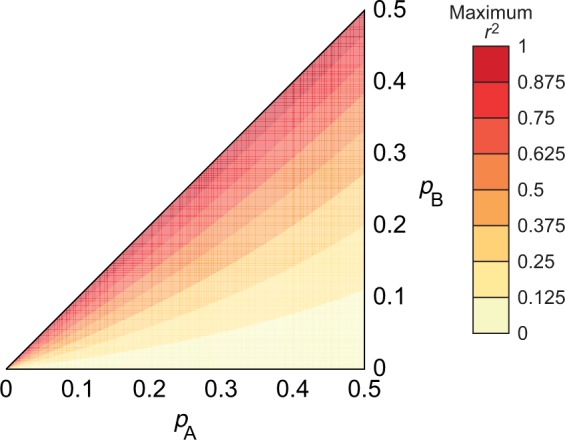
Contour plot of the maximal value of the *r*^2^ LD statistic for a pair of loci. The frequency of *A*, the minor allele of the first locus, is *p_A_*, and the frequency of *B*, the minor allele of the second locus, is *p_B_*, with *p_A_* ≥ *p_B_*. The maximum *r*^2^ is (1 − *p_A_*)*p_B_*/[*p_A_*(1 − *p_B_*)]

*r* = 1 if and only if *p_A_* = *p_B_* = *p_AB_*, and *r* = −1 if and only if *p_A_* = *p_b_* = *p_Ab_*. In both cases, as a function of *p_A_*, *p_B_* and *p_AB_*, the maximum possible value of *r*^2^, or 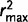, equals 1 if and only if the allele frequencies are equal at the two loci (i.e. {*p_a_*, *p_A_*} = {*p_b_*, *p_B_*}). Without loss of generality, interchanging loci and alleles so that *p_A_* ≤ 1/2, *p_B_* ≤ 1/2 and *p_A_* ≥ *p_B_*, *r*^2^ has a maximum value of (1 − *p_A_*)*p_B_*/[*p_A_*(1 − *p_B_*)] (Fig. 1) [10–12].

As an example, consider two pairs of loci, one with *r^2^* = 1/40 and another with *r*^2 ^= 1/4. It is tempting to conclude that the first pair displays ‘lower LD’ than the second. If the first pair of loci has (*p_A_*,*p_B_*) = (1/3,1/51), then *r*^2^ has a maximum of 1/25, and the observed *r*^2^ of 1/40 is 62.5% of the maximum value. If the second pair of loci has (*p_A_*,*p_B_*) = (1/3,1/5), then *r*^2^ has a maximum of 1/2, and the observed *r*^2^ is only 50% of the maximum. Thus, simply relying on the magnitude of *r*^2^ can fail to provide a complete sense of the nature of LD between a pair of loci.

The dependence of *r*^2^ on allele frequencies affects the search for disease genes in several ways. For example, as discussed below, power to detect a causal variant using a single-nucleotide polymorphism (SNP) marker is related to the value of *r*^2^ between the causal variant and the marker. Thus, if the minor allele frequencies of a SNP marker and a causal SNP are dissimilar, then the power to detect an effect at the marker can be small. As another example, when a marker is reliably detected by GWA studies as associated with disease, one standard method of searching for the causal variant is to look for variants in high LD with the marker according to the *r*^2^ measurement. This approach can suffer when the minor allele frequencies of the marker and the causal variant are dissimilar, as high values of *r*^2^ are then impossible. Methods for accounting for the allele-frequency dependence of *r*^2^ in an association context are now under development. For example, Zhu *et al.* [13] propose that loci whose *r*^2^ values with a disease-associated marker are relatively high compared with their *r*^2^ values with other non-disease-associated markers should be prioritized as candidate disease loci. With increasing interest in rare disease-causing variants—which occupy a different part of the allele-frequency space from the variants that have been of primary interest in most GWA studies to date—efforts to incorporate or circumvent the frequency-dependence of *r*^2^ are sure to continue.

## 2. WINDFALL: EVEN THOUGH THE WORLDWIDE HUMAN POPULATION IS IN THE BILLIONS AND THE HUMAN GENOME CONTAINS MILLIONS OF SNPS, A SAMPLE OF FEWER THAN 1000 PEOPLE IS SUFFICIENT FOR IDENTIFYING MOST OF THE ‘COMMON’ HUMAN GENETIC VARIANTS

Given that the allele frequencies of marker SNPs influence the possibility that they can be used to efficiently detect disease variants of particular frequencies (point 1), it is essential to the GWA program to have a catalog of marker SNPs at known frequencies. The International HapMap Project [14] was launched in 2002 with the aim of facilitating future GWA studies by identifying ‘common’ SNPs in a small number of human populations. These SNPs, typically with a minor allele frequency above 0.05 or 0.1, would then be used to identify a subset of ‘haplotype-tagging’ SNPs that could be genotyped in association studies and tested for disease association. Since it was already predicted that the number of SNP variants would run into the millions, it might seem unsettling that the initial sample size used in the HapMap project was only 269. Even more apparently surprising is that most of the SNP discovery process relied on even smaller panels of individuals.

Straightforward calculations show, however, that most common polymorphisms will be detected as variable even in relatively small samples. Consider a site for which the minor allele has frequency *p* ≤ ½ in a population and the major allele has frequency 1 − *p*. If people are sampled independently at random and each person’s two alleles at the site are independent, then the probability that both allelic types occur at least once in a sample of *n* people is
(1)
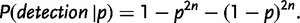



**Figure 2.**
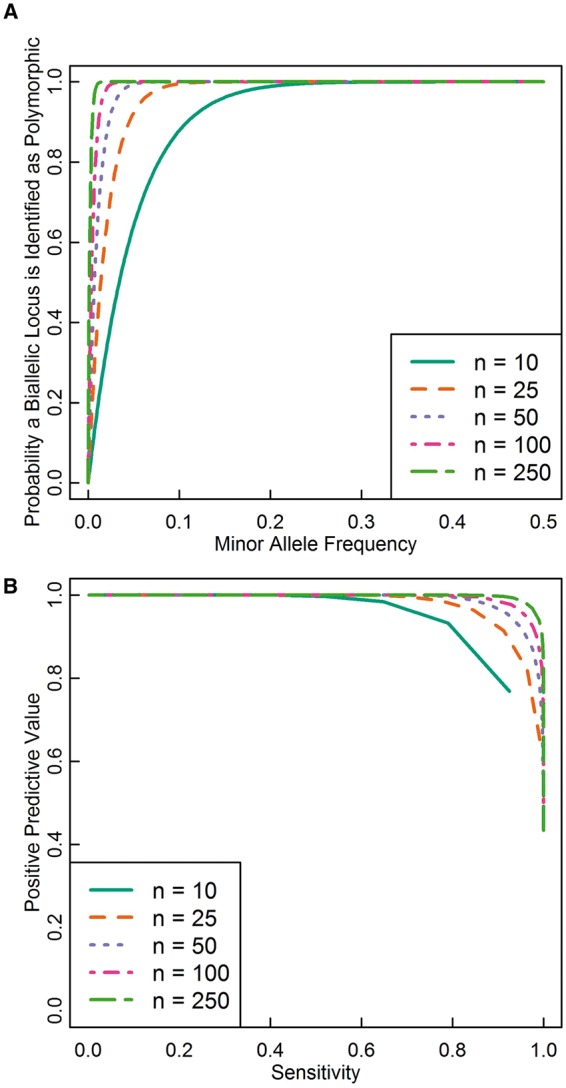
Detection of polymorphic loci. **(A)** The probability that both alleles at a biallelic locus appear in samples of various sizes conditional on minor allele frequency (Equation 1). Most loci with minor allele frequencies of 0.1 or greater appear even in a sample of 10 people (20 chromosomes). **(B)** PPV and sensitivity for detecting loci with minor allele frequencies of 0.05 or greater in various sample sizes. The curve is constructed by calculating the sensitivity and PPV for each choice of *k* (Equations 3 and 4), where *k* is the minimum number of copies of each variant at a biallelic site that must be observed to accept the site into the set of loci with common minor alleles. As *k* increases from 1 to *n*, sensitivity decreases from near 1 to near 0, while PPV increases from its minimum value, which is lower for larger *n*, to near 1. As the sample size grows, choices of *k* that give higher sensitivity and PPV become available. With sample sizes in the low hundreds, it is possible to detect over 90% of loci with minor allele frequency >0.05 while ensuring that fewer than 10% of the identified loci have minor allele frequencies <0.05, assuming that allele frequencies are distributed as predicted by the neutral infinitely-many-sites model with constant population size

This result is obtained by noting that the probability that every observation is of the minor allele is 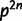 and that the probability that every observation is of the major allele is 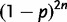. Figure 2A shows the probability that for a locus with a minor allele of a given frequency, both alleles will appear at least once in a sample of a given size. Even small samples identify most loci with polymorphisms of appreciable minor allele frequency.

What might have initially seemed to be a surprisingly small sample size might now appear surprisingly large—if a sample of just 100 people identifies >86.6% of the loci with minor allele frequency 0.02 and >99.99% of loci with minor allele frequency 0.05, then why do we need larger samples?

Consider the situation at the beginning of the HapMap project. Interest was focused on common variants, and one important goal was to catalog common variation in the human genome. If any variable locus that appears in a sample is identified as a SNP, then most common variants will be identified, but some rare variants, which are less useful for haplotype tagging, will also be identified. The degree to which we risk misidentifying loci with rare minor alleles as loci with common minor alleles depends on the distribution of allele frequencies in the population, a consequence of the population’s evolutionary history. The number of variable loci identified at minor allele frequency *p* is proportional to the product of the probability of detecting a variable locus conditional on *p*, given in Equation (1), and the number of loci with minor allele frequency *p*. The more prevalent loci with rare minor alleles are in the population, the more the set of identified variants will contain (unwanted) rare variants.

Population-genetic models of neutral evolution predict that the number of variable loci with minor allele frequency *p* decreases as *p* increases. That is, a large fraction of variable loci will have rare minor alleles. Consider the standard neutral infinitely-many-sites mutation model, which assumes that each new mutation occurs at a previously unmutated locus and that all mutations are selectively neutral. Assuming that mating is random and that the population size is constant across generations, the model predicts that the number of loci with minor allele frequency *p* is proportional to 1/[*p*(1 − *p*)] (e.g. modifying [15], eq. 1.56 by adding the frequencies of loci with derived allele frequencies of *p* and 1 − *p* to get the folded site frequency spectrum; that is, 1/*p* + 1/(1 − *p*) = 1/[*p*(1 − *p*)]). Most new mutations are lost before they can drift to high frequency, and thus, most variable loci have low minor allele frequency.

In the presence of abundant rare variants, researchers who aim to identify only common variants must filter out loci with rare minor alleles. Suppose that we want to identify only loci with minor allele frequency *c* or greater. Eberle and Kruglyak [16] proposed a natural strategy for achieving this goal—namely, to require that each variant at a locus be observed several times, say *k* times, to classify the locus as a candidate haplotype-tagging SNP. Eberle and Kruglyak studied both the probability that a locus is detected as variable given its minor allele frequency and the minor allele frequency distribution of the discovered SNPs. For illustration, we extend their results by deriving the probability that a locus is detected as variable given that its minor allele frequency is greater than some threshold value, as well as the proportion of the discovered SNPs that have minor allele frequencies greater than the threshold value.

Summing over the binomial distribution, if chromosomes are independent, then the probability that each allele at a variable locus with minor allele frequency *p* appears at least *k* times in a sample of *n* people, 1 ≤ *k* ≤ *n*, is
(2)
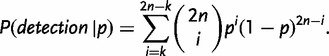



Rare variants are unlikely to appear many times in the sample, so the representation among the identified loci of sites with low minor allele frequency decreases with increasing *k*. The sensitivity for identifying loci with minor allele frequency *c* or more is given by
(3)
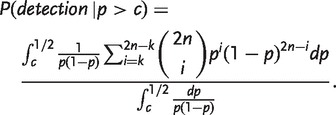



The sensitivity is the probability that each of the two alleles at a locus appears at least *k* times in a sample of *n* people given that the locus has a minor allele frequency of *c* or greater. That is, it is the expected proportion of loci with minor allele frequencies in the desired range that the method will successfully identify.

In contrast, the positive predictive value (PPV) is the probability that a locus has a minor allele frequency of *c* or greater given that each of its two alleles appears *k* or more times in the sample. That is, it is the proportion of loci identified by the procedure that would be expected to actually have minor allele frequencies in the desired range. To calculate the PPV, we apply Bayes’ Theorem and integrate to get
(4)
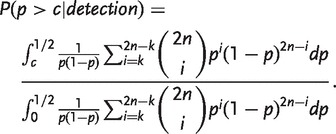



For finite population sizes, the integrals in the expressions for both sensitivity and PPV are replaced by analogous sums.

Figure 2B shows sensitivities and PPVs for various sample sizes drawn from an infinite population, choosing *c* = 0.05 as the minimum desired minor allele frequency. As the sample size increases, it becomes possible to choose *k* such that few loci with minor allele frequencies less than *c* are identified but nearly all loci with minor allele frequencies greater than *c* are identified. For example, with a sample size of 250 people, choosing *k* = 30 identifies >93.3% of loci with minor allele frequency >0.05 and >99.99% of loci with minor allele frequency >0.1, and only 0.7% of the identified loci will have minor allele frequencies <0.05. Thus, under a model of neutral evolution with infinitely-many-sites mutation and constant population size, a sample of a few hundred is large enough to provide a choice of *k* that allows detection of most common variants while screening out most rare variants.

At present, more than 10 years after the launch of the HapMap project, researchers are increasingly interested in the possible role of rare variants in phenotypic variation [17–21], and a current goal is to catalog loci with minor allele frequencies as low as *c* = 0.005 or *c* = 0.001. However, as we approach lower target minor allele frequencies, human evolutionary history complicates the task of specifically identifying loci with minor allele frequencies above a chosen level. In particular, the historical growth of the human population, which violates the standard neutral model, calls for new calculations.

In a growing population, the assumption of constant population size leads to a significant underestimate of the fraction of variable loci whose minor alleles have low frequency. Humans have experienced population growth for many generations, thereby increasing the proportion of rare variants relative to the prediction of the neutral model [18, 22]. When population growth is taken into account, the great majority of variants are predicted to have minor allele frequencies <0.01 [18, 23, 24]. Sequencing studies support this prediction [19, 25], with one study finding that 86% of identified variants had minor allele frequencies <0.005 [21]. This increased prevalence of rare variants decreases the PPV of procedures for detecting loci with common alleles compared with results obtained under constant population size.

Larger samples are then required for meeting the present goal of cataloging loci with low minor allele frequencies, especially if high sensitivity and PPV are required. One study, using a statistical model for allele frequencies with parameters estimated from small sequence data sets, suggested that with a *k* = 1 threshold, achieving 80% sensitivity for loci with a minimum minor allele frequency of 0.001 requires a sample of ∼150 people, and achieving 99% sensitivity requires ∼1000 people [26]. These computations ignore the PPV, which is likely to be low with *k* = 1. The desired sample sizes will be further inflated if *k* is set larger than 1 to filter out loci with minor allele frequencies less than *c*. Regardless of the specific form of the distribution of allele frequencies or the frequencies targeted for ascertainment, the overall strategy outlined here can serve as a guide for understanding the detection of variable loci in a sample.

## 3. PITFALL: IN A CASE-CONTROL STUDY, THE RESULT THAT A SIMPLE 1/*R*^2^ SAMPLE-SIZE INFLATION FACTOR RELATES THE SAMPLE SIZE NEEDED TO DETECT DISEASE ASSOCIATION AT A CAUSAL LOCUS TO THE CORRESPONDING SAMPLE SIZE NEEDED AT A LINKED MARKER LOCUS DEPENDS ON ASSUMPTIONS THAT ARE NOT ALWAYS WARRANTED

In an important analysis of the population genetics of LD in association studies, Pritchard and Przeworski [27] demonstrated that if a disease locus is in LD at level *r*^2 ^= *d* with a marker locus that is otherwise unrelated to disease status, then a 2 × 2 chi-square test for disease association at the marker locus with a sample of size *n/d* has approximately the same power as a test at the true disease locus with sample size *n.* This result, which for diploids assumes that it is the alleles at a locus rather than the diploid genotypes that are tested for association with the phenotype, has influenced both SNP selection and sample size determination in GWA studies [28, 29]. However, relying on this result generates a potential problem: as was discussed most provocatively by Terwilliger and Hiekkalinna [30], the assumptions necessary for the result’s derivation do not always hold, and when they are violated, the properties of power and sample size inflation can be dramatically different. How, then, are we to think about the sample size inflation factor, its underlying assumptions, and their relevance for association studies?

We can build intuition about the sample size inflation factor and its underlying assumptions by considering the population genetics of LD using concepts developed outside genetics, in psychometrics and econometrics. As we will see, Pritchard and Przeworski’s result is closely related to a set of results from psychometric true score theory, and the central complaint of Terwilliger and Hiekkalinna’s [30] provocative reply can be viewed as an application of an econometric viewpoint to the same problem. We start by explaining the relationship between Pritchard and Przeworski’s result and psychometric true score theory. Next, we consider a more general view of measurement error inspired by econometrics, and we explain how Terwilliger and Hiekkalinna’s concern arises from this view.

In psychometric true score theory, it is assumed that the results of a measurement are the sum of a ‘true score’ and a random measurement error [31]. For example, suppose a psychologist asks a participant to solve a puzzle as quickly as possible. We might conceive of the measured time as arising partly from the participant’s true ability to solve the puzzle—which would remain constant if we were to administer equally difficult puzzles in repeated studies—and as partly due to other, random factors. The impact of this second set of factors would be expected to vary across repetitions of the procedure. In classical psychometrics, the component that remains the same across repeated measurements is known as the ‘true score’ and can be viewed as the expectation of the score for the person or other entity being measured [32]. The component that varies across repeated measurements is known as ‘measurement error’. Measurement errors are usually assumed to be independent of true scores of other variables under study and of other measurement errors from separate replicates. Because of this independence, the variance of the observed scores in a population is the sum of the variance of the true scores in the population, or true score variance, and the variance of the measurement errors, or error variance. The reliability of a measurement is defined as the proportion of its observed variance that is true score variance,

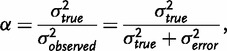

a quantity that also equals the square of the correlation between the observed and true scores.

**Figure 3.**
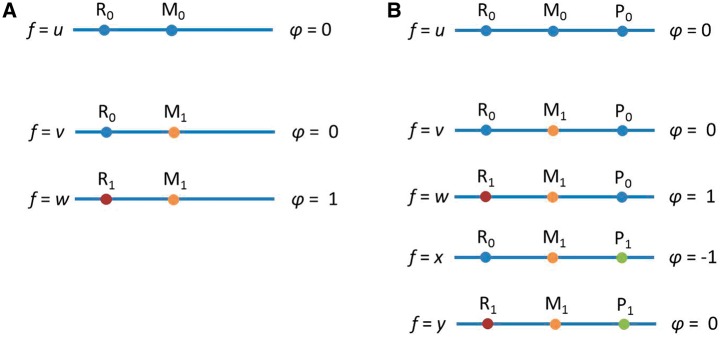
Examples relating to the 1/*r*^2^ sample size inflation factor. **(A)** A situation in which the 1/*r*^2^ sample size inflation factor holds. **(B)** A situation in which it does not generally hold. In both cases, we genotype a neutral marker locus *M*, and the question is by what factor one needs to multiply one’s sample size to detect the relationship between *M*_1_ and the phenotype of interest, *φ*, with the same power that would be obtained if the risk locus, *R*, were genotyped directly. In (A), we have just the marker locus, *M*, and the risk locus, *R*. The alleles at the marker locus do not affect the phenotype *φ*, but at the risk locus, the *R*_1_ allele increases *φ* by one. No recombination events have occurred to separate the loci, so the *R*_1_*/M*_0_ haplotype does not exist. The frequencies (*f*) of the three available haplotypes are *u*, *v* and *w*, as shown. In this case, the 1/*r*^2^ sample size inflation factor holds, where *r*^2^ measures the LD between the risk locus *R* and the marker locus *M*. After conditioning on the allele at the risk locus *R*, the marker locus and the phenotype are independent. In (B), we add a protective locus, *P.* The *P*_0_ allele does not change *φ*, but the *P*_1_ allele decreases *φ* by one. We assume that *P*_1_ co-occurs only with *M*_1_. If either *x* or *y* is positive, then the 1/*r*^2^ sample size inflation factor does not provide the same power for detecting risk at marker locus *M* as if risk locus *R* were genotyped directly, because the association between the marker *M* and the phenotype is no longer due strictly to the risk locus *R* but depends also on the protective locus *P*. If *x* < *w*, then more subjects will be required than would be predicted by the 1/*r*^2^ result. If *x* = *w*, then *M* will not be associated with *φ*, and no number of subjects will provide power greater than the type I error rate. When *x* > *w*, *M*_1_ is associated with lower, rather than higher, values of *φ*, and the direction of the relationship from (A) is reversed. In contrast to (A), when we condition on the allele at the risk locus *R*, the marker locus *M* is still associated with the phenotype because both *M* and the phenotype are associated with the protective locus *P*

To view the problem of identifying disease loci via association studies from a psychometric standpoint, consider a study participant’s allele at a disease locus as a true score, her allele at the marker locus as an observed score, and the *r*^2^ measure of LD as the reliability, the square of the correlation between the observed score and the true score. From this perspective, the 1/*r*^2^ sample-size inflation factor in the association context can be seen as an instance of a class of results on the relationship between the power to reject hypotheses about the true score and the reliability of observed scores. Psychometricians have shown that if the reliability of a measurement is changed from *α* to *α′* as a result of a change in measurement error—that is, holding the true score variance constant—then for a variety of statistical tests, the sample size required to obtain a given power level changes by a factor of *α*/*α′* [33–36]. In the LD setting, error-free measurement of the causal locus has reliability 1, and measurement of the marker has reliability *r*^2^, producing exactly the 1/*r*^2^ inflation factor (Fig. 3A).

Application of Pritchard and Przeworski’s results requires that the assumptions necessary for its derivation are met, or at least that the possibility and potential effects of their violation are understood. As pointed out by Terwilliger and Hiekkalinna [30], several of these assumptions warrant special consideration. One of their most important complaints is that the 1/*r*^2^ inflation factor assumes that the marker locus and disease status are independent after conditioning on the causal locus. If this condition is not met—as can happen, for example, when the marker locus is in LD with more than one causal locus—then either more or fewer subjects might be required. In extreme cases, the causal locus might not be detectable at all.

To understand this caveat, consider the variance of the allelic state at the marker locus that remains after conditioning on the causal locus—the analog of the psychometric error variance. To obtain the 1/*r*^2^ result, we assume that the genetic ‘error variance’ is independent of the phenotype. This corresponds with standard assumptions in psychometrics, where it is assumed that measurement error is independent of the true score and of all other variables under study. Terwilliger and Hiekkalinna’s critique amounts to a comment that measurement errors might in fact be correlated with the dependent variable. In econometrics and other fields that rely largely on observational data, there is a long tradition of considering such possibilities. Measurement errors in the independent variable, regardless of their correlation with the dependent variable, can be considered in the general framework of endogeneity, which arises whenever one or more of the independent variables is correlated with the error term in a regression model [37].

As shown in the Appendix, measurement errors in the independent variable can lead (and usually do lead) to bias in the estimation of the relationship between the independent and dependent variables. When the measurement errors are statistically associated with the dependent variable, the direction and degree of bias are flexible, and in principle, it is possible for the strength of the observed relationship to be inflated, decreased, eradicated or even reversed in direction compared with the true relationship. Naturally, then, when measurement errors in the independent variable are associated with the dependent variable, changes in measurement error are no longer guaranteed to alter required sample sizes by a factor of *α/α′*. Depending on the direction and strength of correlation between the measurement errors and the dependent variable, the required sample size might be larger or smaller than the 1/*r*^2^ factor suggests, or, in extreme cases, the relationship might not be detectable at all.

Consider the case illustrated in Fig. 3B, in which the marker locus is in LD with two loci, one of which has a risk allele and a neutral allele (the risk locus), and the other of which has a protective allele and a neutral allele (the protective locus). It is possible for the observed correlation between the marker locus and the phenotype to take on any value between −1 and 1, depending on the various haplotype frequencies. This is true even when both the LD between the marker locus and the risk locus and the effect of the risk locus on the phenotype are relatively large. For example, when *w* = *x* = ¼ and *u* = ½ (see the figure for notation), the risk locus accounts for 2/3 of the variance in the phenotype, and the *r*^2^ measure of LD between the marker locus and the risk locus is 1/3, but the marker locus is not correlated with the phenotype. The change from the situation in Fig. 3A can be viewed as arising from the fact that the measurement error in Fig. 3B, that is, the variance in the marker locus that is unrelated to the risk locus, is correlated with the phenotype because of its correlation with the protective locus.

Terwilliger and Hiekkalinna [30] claimed that typical misapplications of the 1/*r*^2^ sample-size inflation factor overstate the power to detect disease association. Although the extent to which such overstatements have affected actual association studies has been debated [38–40], a clear conclusion is that it is important to recognize the assumptions that underlie the derivation of sample-size inflation factors. This recognition can assist in analyzing other cases in which imperfect measurement of a causal locus has an effect on association test statistics [41, 42].

## 4. WINDFALL: GENOTYPE IMPUTATION WORKS

Genotype imputation is an application of population genetics that has advanced the recent wave of GWA studies [43–45]. In typical imputation applications, genotypes from study subjects are augmented by data from reference samples that have been fully sequenced or genotyped on a denser collection of markers. The reference data are used to impute genotypes in the study subjects at positions genotyped in the reference sample but not genotyped in the study sample. In an imputation-based association study, positions that are newly imputed in the study sample are tested for disease association using procedures similar to those used at positions for which genotypes have been experimentally measured.

Two aspects of genotype imputation differ substantially from typical imputation problems in survey data and other areas of statistics [46]. First, a variable for which no one in the study has actually been measured can be imputed accurately. Second, the number of variables imputed in any given subject can vastly exceed the number of variables measured. From the perspective of practitioners working in typical missing data settings, the success of genotype imputation might be unexpected: A few hundred thousand variables are measured directly in a sample, and millions of additional variables that were not measured in any of the study subjects can be imputed with mean accuracy well over 90%. Is this not just ‘making up data’?

**Figure 4.**
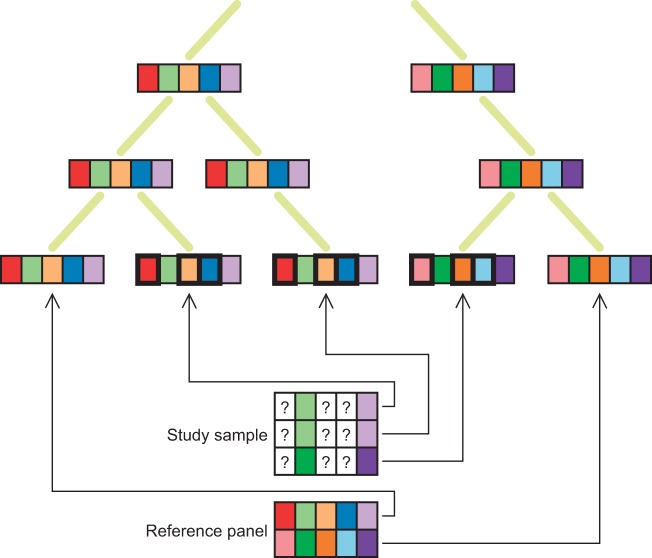
The population-genetic principle underlying genotype imputation. At the bottom of the figure, rows represent haplotypes, and columns represent genomic positions, each with two distinct alleles represented by two colors. Some positions are genotyped only in the reference panel and not in the study sample. Imputation of missing genotypes in the study sample (question marks) is successful because study and reference haplotypes are related by descent, as indicated by the genealogy. Identity of a study haplotype to a reference haplotype at genotyped positions suggests shared descent and therefore identity at intervening ungenotyped positions as well. This matching enables imputation of missing genotypes in the study sample (thick black boxes)

The success of imputation methods is a consequence of the strength of the correlation structure among nearby markers along the genome, which in turn results from the evolutionary descent of genetically similar haplotypes from shared ancestral haplotypes (Fig. 4). If a haplotype in a study sample matches a haplotype in a reference panel at a series of genotyped positions, then it is likely that the study and reference haplotypes are inherited identically by descent from a recent common ancestor. The alternative explanation that identical genotypes were generated by distinct mutational paths becomes increasingly unlikely for long sequences of shared alleles. As a result, the study and reference haplotypes likely share alleles identically by descent at the intervening positions. Those positions can therefore be imputed accurately by copying alleles with the reference haplotype as a template. Although the imputation will be imperfect owing to insufficient sharing between study and reference haplotypes or imperfect choices of templates from the reference panel, studies with numerous algorithms, reference panels and target populations have found that imputation accuracy is often remarkably high [47–52]. Correlations between neighboring sites are so strong that each SNP contains much less than one bit of information when considered in the context of its neighbors, allowing for accurate imputation once enough SNPs are typed.

Upon its introduction, imputation quickly became a routinely used component of the GWA toolbox. Imputed genotypes were quickly incorporated into fine-scale mapping studies and meta-analytic studies that combined different marker sets [44, 45]. In these studies, imputed genotypes can be tested directly for association with the phenotype of interest in the same manner as that used for genotypes that are measured directly. It may seem as though the imputed genotypes, because they are based directly on the measured genotypes, cannot contain any additional information beyond what is available from the measured genotypes themselves. However, in standard association study analysis techniques, each SNP’s association with the phenotype is tested in isolation; including imputed SNPs effectively allows the researcher to test for association between a phenotype and a joint signal from several neighboring loci. This approach boosts power for detecting associations between a phenotype and variability in a particular region of the genome [53, 54].

The utility of genotype imputation increases with the accumulation of population-genetic data: as reference panels gather more individuals and populations, the chance increases that similar haplotypes can be discovered in a reference panel, even for study individuals with unusual haplotypic patterns. This success of imputation represents a milestone in applied population genetics, shifting the focus of genotypic correlations from their use in describing patterns of LD to their use for genotypic prediction.

## 5. (RELATIVE) WINDFALL: POPULATION STRUCTURE IS NOT A SUFFICIENT CONDITION FOR PRODUCTION BY POPULATION STRATIFICATION OF FALSE POSITIVES IN AN ASSOCIATION STUDY

When conducting association studies in structured populations, it is widely recognized that false positive associations between genotypes and phenotypes are systematically produced [55–57]. However, confusion has existed regarding the way in which population structure produces spurious associations. Population stratification—the characteristic of an association study conducted in a structured population that enables production of false positive associations—has been widely conflated with population structure, a mere difference in allele frequencies among subgroups in a population, which can arise when phenomena such as mutation, genetic drift or local selection lead to genetic differences between relatively isolated groups. In a population that consists of distinct subgroups, regardless of whether any phenotypes have been measured, population structure exists when a difference in allele frequencies occurs among the subgroups.

**Figure 5.**
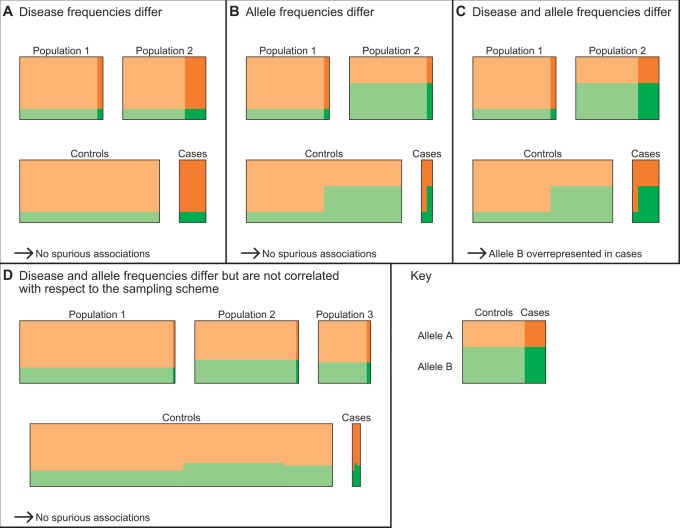
Production of spurious associations in a sample containing individuals from multiple populations. The area of a box is proportional to the frequency represented. **(A)** Disease frequencies differ across the populations, but allele frequencies do not differ, and no spurious associations are produced. **(B)** Allele frequencies at a locus differ across the populations, but disease frequencies do not differ, and no spurious associations are produced. **(C)** Disease and allele frequencies both differ across the populations, and allele *B* is spuriously associated with disease. **(D)** Disease and allele frequencies both differ across the populations, but in a manner that does not satisfy the required condition for production of spurious associations. With *p_i_, q_i_, γ_i_* representing the disease frequency in population *i*, the frequency of allele *B* in population *i*, and the prior probability that a sampled individual is drawn from population *i*, the parameters represented are (*p*_1_,*p*_2_,*q*_1_,*q*_2_,γ_1_,γ_2_) = (1/16, 1/4, 1/6, 1/6, 1/2, 1/2) in A, (1/16, 1/16, 1/6, 7/12, 1/2, 1/2) in B, (1/16, 1/4, 1/6, 7/12, 1/2, 1/2) in C, and following an example of Rosenberg and Nordborg [54], (*p*_1_,*p*_2_,*p*_3_,*q*_1_,*q*_2_,*q*_3_,γ_1_,γ_2_,γ_3_) = (1/100, 1/50, 7/100, 1/4, 3/8, 1/3, 1/2, 1/3, 1/6) in D

In the context of disease–gene association studies, however, the situation is markedly different. In a structured population consisting of two subgroups, population stratification—that is, spurious association produced by population structure—occurs if and only if differences across subgroups exist in both allele frequencies and disease prevalence [58]. The condition of between-subgroup allele frequency difference, or population structure, is necessary for spurious associations to be produced, but it is not sufficient (Fig. 5): a between-subgroup difference in disease prevalence must also occur. More generally, if the structured population contains more than two subgroups that differ in allele frequency, then a disease prevalence difference across subgroups is not even sufficient to produce spurious associations: it is further required that a correlation exist between allele frequencies and disease prevalence with respect to the sampling scheme across subgroups [59, 60]. More formally, considering a collection of populations 1, 2, … , *I* and denoting the disease prevalence in population *i* by *p_i_*, the frequency of a specific allele in population *i* by *q_i_*, and the prior probability that an individual in the structured population is drawn from population *i* by γ*_i_*, production of spurious associations with the allele of interest requires that 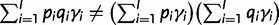.

Why is population structure insufficient for production of spurious associations? Consider the simplest case of two subgroups. If disease prevalence is identical between the populations, then neither subgroup is overrepresented among either cases or controls (Fig. 5). If members of the two subgroups are present in equal proportions in the affected and control samples, then for loci with no true association with disease, the allele frequency differences between the subgroups do not lead to allele frequency differences between the affected and control samples.

The dependence of the severity of population stratification on disease prevalence differences and allele frequency differences is captured in a non-negative parameter δ. This parameter measures the amount of population stratification for a given marker and disease in an association setting [61, 62], taking the disease prevalence into account. Thus, in the same structured population, the magnitude of population stratification is variable across different phenotypes. The existence of population structure is likely to be accompanied by at least some non-trivial differences in phenotypic prevalences, and as discussed in the next section, these differences become more important and potentially problematic as sample sizes increase. Still, it is important to recognize the distinction between the population-genetic phenomenon of population structure and the association-study concept of population stratification: the existence of structure does not on its own imply that spurious associations will be widespread for a given phenotype. It may even be helpful to view population stratification as simply one of many potential sources of confounding in association studies, as we suggest in the next section. Indeed, current approaches are increasingly incorporating mixed models that can account for many types of confounding, including different forms of population structure, differences in relatedness within populations and environmental effects [63].

## 6. PITFALL: AS A RESULT OF POPULATION-GENETIC PHENOMENA, MARKERS THAT ARE CONSISTENTLY ASSOCIATED WITH DISEASE IN MULTIPLE STUDIES ARE NOT GUARANTEED TO BE CAUSAL OR IN CLOSE LINKAGE WITH CAUSAL MUTATIONS

In general, the alternative hypothesis is typically not identical with the scientific proposition under study, meaning that if the null hypothesis is false, several explanations are available in addition to the one that the study was designed to examine. For clinical trials, tactics such as randomization and blinding limit the number of tenable alternative explanations. In GWA studies, however, the population genetics of LD produces several ways in which a non-causal site can be reliably associated with a phenotype in the population, even when it is not linked to the causal loci or when the causal loci all have smaller or even zero association with the phenotype. We refer to these stably replicable but etiologically misleading associations as spurious associations. Spurious associations can be contrasted with genuine associations, or associations between phenotypes and variants that are either causal or in close linkage with causal associations, and with stochastic associations, or associations between phenotypes and non-causal variants that arise in individual studies due to type I error. Spurious associations are like genuine associations in that they are true features of the population, but they are like stochastic associations in that they provide no insight into etiology and can divert attention from more productive genomic regions.

As we saw in the previous section, population stratification is one way in which spurious associations can arise. Platt *et al.* [64] note three mechanisms by which spurious associations can occur, the first of which encompasses population stratification: correlation of the non-causal site with unlinked causal sites, multiplicity of causal factors and disease models involving interaction either between genetic factors or between genetic and environmental factors. We consider each of these possibilities in turn.

First, it is possible that a non-causal locus can be correlated with other loci even in the absence of linkage between the loci. This does not occur in an unstructured, infinite population at Hardy–Weinberg equilibrium, but there are several ways it can happen in real populations, including genetic drift, inbreeding and gene conversion [65]. For example, if two subpopulations are reproductively isolated, then their allele frequencies drift independently of each other, leading to allele frequency differences across the genome. If the two subpopulations are then considered as one population, there will be correlations between loci that reflect the differing ways drift has operated in the two subpopulations. This example is well-known and is one form of the population structure described in the previous section. A version of the phenomenon can also occur with the case-control ascertainment process, where separate subpopulations of cases and controls act similarly to partially isolated subpopulations, leading to correlations between unlinked loci in a sample even when the loci are not correlated in the population [66, 67]. Platt *et al.* give an additional, more complex hypothetical example involving pleiotropy and local selection. Consider two unlinked polymorphisms, one that affects both skin and eye color and another that affects only skin color. Suppose further that skin color is under local selection, such that in some geographic areas, darker skin is advantageous. In these areas, the frequency of alleles contributing to darker skin will increase at both loci, which will lead to correlation between loci when the whole population is considered. Under these circumstances, adequately powered studies would detect an association between eye color and the locus affecting only skin color. The two loci develop a correlation because selection elevates the alleles with the favored effect on skin color in a local segment of the population, and through this induced correlation, any phenotype that is influenced by only one of the two loci will have an association with both loci.

**Figure 6.**
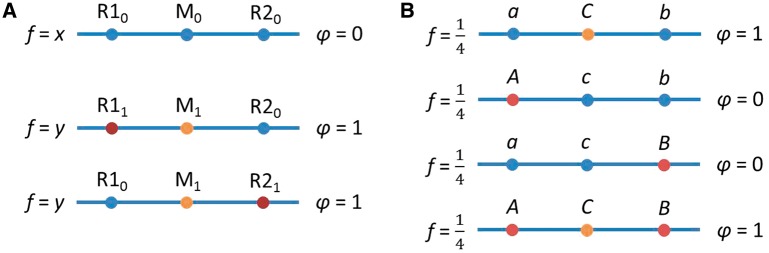
Two types of spurious association outlined by Platt and colleagues [64]. **(A)** The marker locus (M) is in LD with two risk loci (R1 and R2). The presence of a risk allele at either risk locus adds one to the phenotype. The case in which an individual has risk alleles at both loci, and thus the phenotype value is 2, is here assumed not to exist in the population, but the conclusion remains similar when this assumption is relaxed. In this example, the marker locus is perfectly correlated with the phenotype, but the actual causal loci have smaller positive correlations with the phenotype. **(B)** The *A/a* and *B/b* loci epistatically determine the phenotype, and the *C/c* locus is a non-causal marker. In this extreme example, the marker is perfectly correlated with the phenotype, but both causal loci are uncorrelated with the phenotype

Second, non-causal sites can also be associated with phenotypes when phenotypes are influenced by multiple causal factors. A non-causal marker that is correlated with two causal factors can be more strongly associated with the phenotype than either of the causal factors is, especially when the causal factors are rare [64]. As an example, take the situation sketched in Fig. 6A. A non-causal marker locus has an allele with frequency *2y*, where *y* is a positive number <1/2. The haplotypes that include this allele always include exactly one risk allele that appears at one of two nearby risk loci, each of which carries a risk allele with frequency *y*. If the phenotype of interest is dictated by the number of risk alleles, then the marker allele will be perfectly associated with the phenotype (*r^2^* = 1), but the two causal loci will each be less strongly associated with disease status:

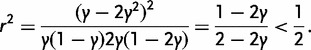

These first two possibilities—correlation with unlinked markers and multiplicity of causal factors—can lead to strong spurious associations between phenotypes and non-causal markers even when it is assumed that causal factors influence phenotypes only in an additive way. It is problematic that the strongest association with the phenotype might come from a non-causal site, but all is not lost: the causal loci will also have non-zero associations with the phenotypes they influence. However, if these assumptions are relaxed to allow interaction of causal factors in the form of either epistasis or gene–environment interaction, then non-causal markers might be associated with the phenotype even while the true causal factors are not. Consider the haploid example shown in Fig. 6B. There are two causal loci, one with alleles *A* and *a*, and the other with alleles *B* and *b*. These sites influence a disease phenotype in an epistatic way, such that an individual with genotype *AB* or *ab* will develop the disease. Suppose that 1/4 of the population has genotype *AB*, 1/4 has *Ab*, 1/4 has *aB* and 1/4 has *ab*. Furthermore, suppose that at a non-causal site, the half of the population with genotypes *AB* or *ab* has one allele *C*, whereas the rest of the population has another allele *c*. In this case, neither of the true causal loci is associated with the disease (*r^2^* = 0), but the non-causal marker is in perfect association with it (*r^2^* = 1). Thus, in models allowing for epistasis, causal loci might not be associated with disease even while non-causal loci have a strong disease association. The interaction need not even be a genetic interaction; the example works in the same way if *B* and *b* are viewed as the possible types of a binary environmental character.

In each of these examples, an association exists at the population level between a marker unconnected to disease and potentially not linked along the genome to causal loci. The associations are not mere stochastic associations due to type I error but rather are true features of the population. Though the null hypothesis of no association between genotypes and phenotypes is in fact false, spurious associations divert attention from the genuine, causally informative associations that interest investigators. Because increases in sample size increase the power to identify both genuine and spurious associations [68], a repeatable, robust association between a locus and a disease in well-powered, well-designed studies need not imply that the locus being examined will be related to the disease, nor need it even imply that the true causal loci will be detectable via an intensive search of the surrounding genomic region.

The extent to which the associations observed in GWA studies are due to factors other than individual, closely-linked causal variants is not known. Dickson *et al.* [69] argued by simulation that some GWA signals might be ‘synthetic associations’—associations occurring between markers and disease as a byproduct of multiple, potentially distant, rare, disease-causing variants. This possibility is a special case of the second source of spurious GWA results described by Platt *et al.*, multiplicity of causal factors, and it underlies attempts to estimate the prevalence of rare variants as sources of GWA signals at more common variants [70, 71]. Synthetic associations are a plausible factor in contributing to at least some GWA signals, but their overall importance in explaining GWA signals is unclear [72–74]. As in the case of the sources of spurious signals classified by Platt *et al.*, they are theoretically possible, but their prevalence is unknown. Without precise knowledge about the prevalence of synthetic associations, it is useful to remain vigilant about their possibility. Continuing increases in sample size and advances in statistical tests for associations between rare variants and disease [75, 76] will further enhance direct analyses of rare variants that could generate synthetic associations. These developments will likely yield refined association signals and reduce the amount of research effort targeted at loci that might be synthetically rather than genuinely related to disease.

## 7. PITFALL: BECAUSE HUMANS SHARE EVOLUTIONARY HISTORY, SEPARATE GENOME-WIDE ASSOCIATION SAMPLES ARE NOT FULLY INDEPENDENT

One of the comforts provided by frequentist hypothesis testing is the freedom to choose the false positive rate. For example, if we run a randomized clinical trial of a medication, and if our statistical model accurately describes the experimental situation, then we can specify the probability of concluding that the null hypothesis is false given that it is actually true. Powerfully, this principle applies to replications as well, multiplying the false positive rate across studies. For example, if we run three independent clinical trials of separate patients and set the false positive rate at 0.05 each time, then the probability of rejecting the null hypothesis in all three trials given that it is in fact true is only 0.05^3^, or 0.000125. This situation leads us to be increasingly confident that null hypotheses that are repeatedly rejected are in fact false, provided that the models used are appropriate and that studies are well-conducted.

Unfortunately, the happy situation of the clinical trial does not translate entirely intact to the GWA study. Shared evolutionary history implies that separate samples are not genetically independent. Two distinct sets of people from one population who are enrolled in separate GWA studies are likely to share at least some of their recent ancestors. Consequently, some of the mutations and recombination events that led to a (potentially spurious) association between a variant and a phenotype in one study might have also occurred in the ancestries of people from the second study. Thus, the outcomes of the two studies will be positively correlated, and the probability of rejecting the null hypothesis in both studies given that the null hypothesis is true will be greater than the square of the chosen type I error rate. Repeated replications of results therefore provide weaker evidence for the validity of the association than might be expected if the studies were truly independent.

Working under the assumption that the null hypothesis is false, Rosenberg and VanLiere [77] used coalescent simulation to show that the probability of rejecting the null hypothesis separately in each of a pair of studies is larger than the probability expected under the hypothesis that the two studies are independent, observing a ‘pseudoreplication’ effect under a variety of conditions on population history and disease models. It is important to note that Rosenberg and VanLiere examined power rather than type I error rates, assuming the null hypothesis was false rather than true. It remains to examine the extent to which simple type I errors as well as misleading associations in the framework of Platt *et al.* [64] can also be pseudoreplicated. The magnitude of the pseudoreplication effect in actual studies is unknown and might be small; even so, pseudoreplication strikingly illustrates the shift in perspective that comes from thinking of association studies population genetically. To the extent that the samples are related by descent, results from separate association studies provide less information than they would were the samples truly independent.

## CONCLUSIONS

Unlike linkage mapping, which utilizes co-transmission of disease with marker alleles in families as a tool for identifying trait loci, the value of association mapping traces fundamentally to the population-genetic properties of study populations. The evolutionary history of populations provides the basis by which associations between genotypes and traits are generated and maintained, and it informs the application and interpretation of statistics used to discover these associations. Consequently, core principles of population genetics provide a bedrock that will always underlie association studies, regardless of what shape new methods and technologies take. Our examples suggest a set of major features of association study design and interpretation on which population genetics can usefully comment:

1. *Population-genetic thinking explains the sources and types of confounding expected in association studies (Points 3, 5, 6 and 7)*. Confounding is a major focus of epidemiology in general, but the correlation structure of genetic data generates distinctive forms of genetic confounding. Individual genetic variants are inherited in large pieces of DNA containing many other variants, and they spread through populations in ways influenced by selection, migration, assortative mating and other evolutionary forces. These phenomena lead to genetic confounds, and they are fundamental topics in population genetics. Population genetics, then, is well-positioned to answer questions about confounding in association studies.
2. *Population-genetic thinking clarifies the relationships between typed variants and causal variants in association studies (Points 1, 3 and 6).* In association studies, the typed genetic variants are not necessarily expected to be the causal variants themselves. Under what circumstances will typed variants reveal the true causal variants, and when will they fail to do so? What will be the size and direction of an observed association between a phenotype and a tagging variant near a causal variant? The answers to these questions depend on the disease model, the statistical procedures used and the distributions of and patterns of associations between genetic variants and environmental factors in the populations under study. Population genetics contributes to tools that can integrate these varied considerations.
3. *Population-genetic thinking reveals the advantages and disadvantages of particular strategies for tracking genetic variation in association studies (Points 1, 2, 3, 4 and 6)*. In association studies, one must decide how to identify, select and measure genetic variants. Which variants should be chosen? How frequent should they be, and where should they be positioned? Can sets of variants be related to each other? How should studies for identifying variants be designed? Must all variants be measured directly, or can some be imputed? If imputation is used, what kinds of reference panels should be constructed? Careful consideration of LD, allele frequency distributions and population structure—again, all core topics in population genetics that are best understood by taking an evolutionary view—is fundamental to addressing these questions.

In sum, application of population-genetic principles can help to avoid pitfalls and to understand windfalls in association studies, and as association mapping continues, it will be partly through thoughtful investigations of population-genetic and evolutionary processes and their effects on patterns of variation that further advances will be possible.

## GLOSSARY TERMS

**Locus.** A genetic locus is a specific site along a chromosome. Loci at which multiple allelic types exist in a population are termed polymorphic.

**Linkage disequilibrium.** Non-random association of alleles at different genetic loci. Suppose we have two loci, one with alleles *A* and *a*, and another with alleles *B* and *b*. Let *p_A_* be the frequency of allele *A*, *p_B_* be the frequency of allele *B* and *p_AB_* be the joint frequency of *A* and *B*, or the probability of carrying both alleles *A* and *B* on a haploid copy of the genome*.* The two loci are said to be in LD if and only if *p_A_p_B_* ≠ *p_AB_*. One important factor maintaining LD is linkage, which occurs when loci are physically close together on a chromosome and thus less likely to be separated during transmission from parent to offspring. However, the presence of LD between loci does not guarantee that the loci are linked, nor are linked loci necessarily in LD [65].

**Single-nucleotide polymorphism.** A single nucleotide position in the genome at which allelic differences occur in a population. In principle, it is possible for a SNP to have two, three or four alleles, but almost all SNPs with common alleles are biallelic. We assume here that SNPs are biallelic.

**Association study.** A type of study in which sites in the genomes of samples of unrelated individuals are scanned for statistical association with a phenotype. In a GWA study, a set of hundreds of thousands or millions of SNPs drawn from across the genome is genotyped, and these SNPs are tested for association with the phenotype. The SNPs themselves might not be causally related to the disease, but might instead be in LD with, and hopefully linked to, the causal mutations.

**Minor allele.** At a locus with two alleles, the minor allele is the one that is less frequent in the population.

**Haplotype.** A haplotype is a combination in an individual of the allelic types at a set of loci. The loci that contribute to a haplotype lie on the same chromosome.

## APPENDIX: MEASUREMENT ERROR AND INDIRECT ASSOCIATION TESTING

In this appendix, we give a description of the bias resulting from measurement error in a simplified setting. For illustrative purposes, we consider the case of least-squares linear regression with no intercept, assuming the variables have been scaled to have expectations of zero. Similar calculations could be performed for other analysis frameworks.

Suppose that the dependent variable *Y* is a linear function of *X*, *U* and *ε*, which are mutually independent random variables with expectations equal to zero and finite variance. That is, for all *i*,

In the association study setting, we can view *Y* as a phenotype of interest, *X* as the allelic state at a causal locus and *U* as representing the variability that remains at a marker locus after conditioning on *X*. That is, we can think of a marker locus in LD with *X* as *X** = *X* + *U.*

Our goal is to estimate  from data. Suppose first that we can measure *X* without error. The least-squares estimator of  is the choice of  that minimizes . This quantity is minimized by setting  . The expectation of the least-squares estimator when *X* is measured without error is

Thus, when *X* is measured without error, the least-squares estimator of  is unbiased.

In contrast, consider what happens when *X* is measured with error equal to *U*. That is, instead of measuring each , we observe . When , the measurement errors are independent of the dependent variable, corresponding to the standard psychometric assumptions discussed in the main text. When , the measurement errors are correlated with the dependent variable. The reliability of  as a measurement of  is .

When we minimize , we obtain . The expectation of  is then

The first step follows from the mutual independence of *X*, *U* and *ε*, combined with the fact that their expectations are zero, and the second step follows from the fact that , so . Thus, if the measurement errors  have non-zero variance, then the least-squares estimate of the slope  is biased when . The expression inside the expectation is positive when the measurement errors have non-zero variance, so when ,  is biased upward as an estimate of , and when ,  is biased downward. Note that the expression inside the expectation is the plug-in estimator for the proportion of variance in the observed independent variable that is attributable to measurement error, or one minus the reliability, . The plug-in estimator is asymptotically consistent. Thus, when the sample size is large, .

This result sheds light on the apparent disagreement of Terwilliger and Hiekkalinna [26] with the work of Pritchard and Przeworski [23]. Pritchard and Przeworski’s results are derived assuming that the marker locus and the phenotype are independent after conditioning on the causal locus, which, in the model used in this appendix, amounts to assuming . When , the observed effect size is biased downward to an extent depending directly on , where  is equal, in the association study context, to the  measurement of LD between the marker locus and the causal locus. In this model, Terwilliger and Hiekkalinna’s principal complaint is equivalent to noting that  is not guaranteed to equal 0, and when it does not, Pritchard and Przeworski’s results no longer hold.

## References

[1] Devlin B, Risch N (1995). A comparison of linkage disequilibrium measures for fine-scale mapping. Genomics.

[2] Kruglyak L (2008). The road to genome-wide association studies. Nat Rev Genet.

[3] Collins FS, Guyer MS, Chakravarti A (1997). Variations on a theme: cataloging human DNA sequence variation. Science.

[4] Risch N, Merikangas K (1996). The future of genetic studies of complex human diseases. Science.

[5] Lewontin RC (1964). The interaction of selection and linkage. I. General considerations; heterotic models. Genetics.

[6] Lewontin RC, Kojima K-I (1960). The evolutionary dynamics of complex polymorphisms. Evolution.

[7] Hedrick PW (1987). Gametic disequilibrium measures: proceed with caution. Genetics.

[8] Hudson RR, Balding DJ, Bishop M, Cannings C (2001). Linkage disequilibrium and recombination. Handbook of Statistical Genetics.

[9] Weir BS (1996). Genetic Data Analysis 2: Methods for Discrete Population Genetic Data.

[10] Eberle MA, Rieder MJ, Kruglyak L (2006). Allele frequency matching between SNPs reveals an excess of linkage disequilibrium in genic regions of the human genome. PLoS Genet.

[11] VanLiere JM, Rosenberg NA (2008). Mathematical properties of the r^2^ measure of linkage disequilibrium. Theor Popul Biol.

[12] Wray NR (2005). Allele frequencies and the r^2^ measure of linkage disequilibrium: impact on design and interpretation of association studies. Twin Res Hum Genet.

[13] Zhu Q, Ge D, Heinzen EL (2012). Prioritizing genetic variants for causality on the basis of preferential linkage disequilibrium. Am J Hum Genet.

[14] The International HapMap Consortium (2003). The International HapMap Project. Nature.

[15] Ewens WJ (2004). Mathematical Population Genetics: I. Theoretical Introduction.

[16] Eberle MA, Kruglyak L (2000). An analysis of strategies for discovery of single-nucleotide polymorphisms. Genet Epidemiol.

[17] Cirulli ET, Goldstein DB (2010). Uncovering the roles of rare variants in common disease through whole-genome sequencing. Nat Rev Genet.

[18] Keinan A, Clark AG (2012). Recent explosive human population growth has resulted in an excess of rare genetic variants. Science.

[19] Nelson MR, Wegmann D, Ehm MG (2012). An abundance of rare functional variants in 202 drug target genes sequenced in 14,002 people. Science.

[20] Siva N (2008). 1000 Genomes project. Nat Biotech.

[21] Tennessen JA, Bigham AW, O’Connor TD (2012). Evolution and functional impact of rare coding variation from deep sequencing of human exomes. Science.

[22] Slatkin M, Hudson RR (1991). Pairwise comparisons of mitochondrial DNA sequences in stable and exponentially growing populations. Genetics.

[23] Coventry A, Bull-Otterson LM, Liu X (2010). Deep resequencing reveals excess rare recent variants consistent with explosive population growth. Nat Commun.

[24] Durrett R, Limic V (2001). On the quantity and quality of single nucleotide polymorphisms in the human genome. Stoch Proc Appl.

[25] Abecasis GR, Auton A, Brooks LD (2012). An integrated map of genetic variation from 1,092 human genomes. Nature.

[26] Ionita-Laza I, Lange C, Laird NM (2009). Estimating the number of unseen variants in the human genome. Proc Natl Acad Sci USA.

[27] Pritchard JK, Przeworski M (2001). Linkage disequilibrium in humans: models and data. Am J Hum Genet.

[28] Carlson CS, Eberle MA, Rieder MJ (2004). Selecting a maximally informative set of single-nucleotide polymorphisms for association analyses using linkage disequilibrium. Am J Hum Genet.

[29] Jorgenson E, Witte JS (2006). Coverage and power in genomewide association studies. Am J Hum Genet.

[30] Terwilliger JD, Hiekkalinna T (2006). An utter refutation of the “fundamental theorem of the HapMap”. Eur J Hum Genet.

[31] Nunnally J (1978). Psychometric Theory.

[32] Lord FM, Novick MR (1968). Statistical Theories of Mental Test Scores.

[33] Subkoviak MJ, Levin JR (1977). Fallibility of measurement and the power of a statistical test. J Educ Meas.

[34] Sutcliffe J (1958). Error of measurement and the sensitivity of a test of significance. Psychometrika.

[35] Williams RH, Zimmerman DW (1989). Statistical power analysis and reliability of measurement. J Gen Psychol.

[36] Williams RH, Zimmerman DW, Zumbo BD (1995). Impact of measurement error on statistical power: review of an old paradox. J Exp Educ.

[37] Greene WH (2012). Econometric Analysis.

[38] Bochdanovits Z, Heutink P, van der Vaart A (2008). Empirical assessment of the validity of the ‘fundamental theorem of the HapMap’ in the light of ‘cryptic’ tagging of multiple susceptibility loci. Eur J Hum Genet.

[39] Moskvina V, O'Donovan MC (2007). Detailed analysis of the relative power of direct and indirect association studies and the implications for their interpretation. Hum Hered.

[40] Thomas DC, Stram DO (2006). An utter refutation of the “Fundamental Theorem of the HapMap” by Terwilliger and Hiekkalinna. Eur J Hum Genet.

[41] Huang L, Wang C, Rosenberg NA (2009). The relationship between imputation error and statistical power in genetic association studies in diverse populations. Am J Hum Genet.

[42] Kang SJ, Gordon D, Finch SJ (2004). What SNP genotyping errors are most costly for genetic association studies?. Genet Epidemiol.

[43] Halperin E, Stephan DA (2009). SNP imputation in association studies. Nat Biotechnol.

[44] Li Y, Willer C, Sanna S (2009). Genotype imputation. Annu Rev Genomics Hum Genet.

[45] Marchini J, Howie B (2010). Genotype imputation for genome-wide association studies. Nat Rev Genet.

[46] Little RJA, Rubin DB (2002). Statistical Analysis with Missing Data.

[47] Huang L, Jakobsson M, Pemberton TJ (2011). Haplotype variation and genotype imputation in African populations. Genet Epidemiol.

[48] Huang L, Li Y, Singleton AB (2009). Genotype-imputation accuracy across worldwide human populations. Am J Hum Genet.

[49] Nothnagel M, Ellinghaus D, Schreiber S (2009). A comprehensive evaluation of SNP genotype imputation. Hum Genet.

[50] Shriner D, Adeyemo A, Chen G (2010). Practical considerations for imputation of untyped markers in admixed populations. Genet Epidemiol.

[51] Yu Z, Schaid DJ (2007). Methods to impute missing genotypes for population data. Hum Genet.

[52] Zhang B, Zhi D, Zhang K (2011). Practical consideration of genotype imputation: sample size, window size, reference choice, and untyped rate. Stat Interface.

[53] Marchini J, Howie B, Myers S (2007). A new multipoint method for genome-wide association studies by imputation of genotypes. Nat Genet.

[54] Spencer CCA, Su Z, Donnelly P (2009). Designing genome-wide association studies: sample size, power, imputation, and the choice of genotyping chip. PLoS Genet.

[55] Lander ES, Schork NJ (1994). Genetic dissection of complex traits. Science.

[56] Price AL, Zaitlen NA, Reich D (2010). New approaches to population stratification in genome-wide association studies. Nat Rev Genet.

[57] Ziv E, Burchard EG (2003). Human population structure and genetic association studies. Pharmacogenomics.

[58] Pritchard JK, Rosenberg NA (1999). Use of unlinked genetic markers to detect population stratification in association studies. Am J Hum Genet.

[59] Gorroochurn P, Hodge SE, Heiman G (2004). Effect of population stratification on case-control association studies. II. False-positive rates and their limiting behavior as number of subpopulations increases. Hum Hered.

[60] Rosenberg NA, Nordborg M (2006). A general population-genetic model for the production by population structure of spurious genotype–phenotype associations in discrete, admixed or spatially distributed populations. Genetics.

[61] Gorroochurn P, Heiman GA, Hodge SE (2006). Centralizing the non-central chi-square: a new method to correct for population stratification in genetic case-control association studies. Genet Epidemiol.

[62] Gorroochurn P, Hodge SE, Heiman GA (2007). A unified approach for quantifying, testing and correcting population stratification in case-control association studies. Hum Hered.

[63] Vilhjálmsson BJ, Nordborg M (2013). The nature of confounding in genome-wide association studies. Nat Rev Genet.

[64] Platt A, Vilhjálmsson BJ, Nordborg M (2010). Conditions under which genome-wide association studies will be positively misleading. Genetics.

[65] Slatkin M (2008). Linkage disequilibrium—understanding the evolutionary past and mapping the medical future. Nat Rev Genet.

[66] Pirinen M, Donnelly P, Spencer CC (2012). Including known covariates can reduce power to detect genetic effects in case-control studies. Nat Genet.

[67] Zaitlen N, Pasaniuc B, Patterson N (2012). Analysis of case–control association studies with known risk variants. Bioinformatics.

[68] Pritchard JK, Donnelly P (2001). Case–control studies of association in structured or admixed populations. Theor Popul Biol.

[69] Dickson SP, Wang K, Krantz I (2010). Rare variants create synthetic genome-wide associations. PLoS Biol.

[70] Anderson CA, Soranzo N, Zeggini E (2011). Synthetic associations are unlikely to account for many common disease genome-wide association signals. PLoS Biol.

[71] Orozco G, Barrett JC, Zeggini E (2010). Synthetic associations in the context of genome-wide association scan signals. Hum Mol Genet.

[72] Goldstein DB (2011). The importance of synthetic associations will only be resolved empirically. PLoS Biol.

[73] Visscher PM, Goddard ME, Derks EM (2012). Evidence-based psychiatric genetics, AKA the false dichotomy between common and rare variant hypotheses. Mol Psychiatry.

[74] Wang K, Dickson SP, Stolle CA (2010). Interpretation of association signals and identification of causal variants from genome-wide association studies. Am J Hum Genet.

[75] Listgarten J, Lippert C, Kang EY (2013). A powerful and efficient set test for genetic markers that handles confounders. Bioinformatics.

[76] Wu MC, Lee S, Cai T (2011). Rare-variant association testing for sequencing data with the sequence kernel association test. Am J Hum Genet.

[77] Rosenberg NA, VanLiere JM (2009). Replication of genetic associations as pseudoreplication due to shared genealogy. Genet Epidemiol.

